# Acute Effects of Blood Flow Restriction Training on Movement Velocity and Neuromuscular Signal during the Back Squat Exercise

**DOI:** 10.3390/jcm12144824

**Published:** 2023-07-21

**Authors:** Manuel García-Sillero, Sergio Maroto-Izquierdo, María Galván-García, Javier Benitez-Porres, Salvador Vargas-Molina, Jose Manuel Jurado-Castro

**Affiliations:** 1 Physical Education and Sport, Faculty of Medicine, EADE-University of Wales Trinity Saint David, 29018 Malaga, Spain; 2 Department of Health Sciences, European University Miguel de Cervantes (UEMC), 47012 Valladolid, Spain; 3 Physical Education and Sport, Faculty of Medicine, University of Málaga, 29016 Málaga, Spain; 4 Metabolism and Investigation Unit, Maimonides Biomedical Research Institute of Cordoba (IMIBIC), Reina Sofia University Hospital, University of Cordoba, 14004 Cordoba, Spain; 5 CIBER Fisiopatología de la Obesidad y Nutrición (CIBEROBN), Instituto de Salud Carlos III, 28029 Madrid, Spain; 6 Ciencias De La Actividad Física y El Deporte, Escuela Universitaria de Osuna (Centro Adscrito a la Universidad de Sevilla), 41640 Osuna, Spain

**Keywords:** neuromuscular performance, blood flow restriction, occlusion, resistance training, velocity, EMG

## Abstract

The aim of this study was to verify the effects of blood flow restriction on movement velocity and muscle activity during the back squat exercise. Methods: Twenty-four university students participated in this study. In two randomized sessions 72 h apart, participants performed a 4-set protocol consisting of 30-15-15-15 repetitions performed at 30% of their one-repetition maximum in the back squat exercise. In both sessions, neuromuscular function was monitored by surface electromyography (EMG) and movement velocity (mean propulsive velocity (MPV), peak concentric velocity (Vmax), and the effort index (EI)). Blood flow restriction (BFR) was applied during exercise in one of the experimental sessions with 80% of full arterial occlusion pressure over lower limbs. Results: The BFR condition showed higher (*p* < 0.05) EI, peak, and rooted mean square normalized EMG in Set 1 compared to Set 2. Similar MPV and Vmax were observed in each set for both the BFR and control conditions. No significant differences were observed between conditions in any set. Conclusions: BFR did not imply changes in neuromuscular performance during low-intensity resistance training, but it might induce greater intra-series velocity loss and less excitation of the muscles involved.

## 1. Introduction

The search for efficiency in the field of training and health is one of the goals of trainers and therapists. New methods and technologies are constantly being applied for this purpose. In recent years, blood flow restriction (BFR) training (BFRT) has been studied for its effects in different populations [1,2]. BFRT is based on low-intensity (usually between 20 and 50% of one-repetition maximum (1-RM)) exercise performed with local hypoxia induced by an inflatable tourniquet located on the proximal limb, which restricts arterial inflow (50–80% commonly) and venous return from the extremity [3]. BFRT is an interesting alternative to high-intensity resistance training for improving muscle strength [4].

Regarding the vascular system, the effects found in the recent literature on the subject showed more significant effects on flow-mediated dilation (FMD) (*p* = 0.002) and improved production of the major angiogenic biomarker vascular endothelial growth factor after BFR compared to non-BFR training. The analysis of pulse wave velocity, ankle–brachial index, systolic blood pressure, and heart rate showed no significant difference in changes between BFR and non-BFR exercise [5].

BFR applied with moderate vascular occlusion has been proven to enhance muscle hypertrophy with low exercise intensity (i.e., 20% of 1-RM) [6]. The scientific literature also supports that BFRT can lead to muscle strength improvements in healthy participants [7]. In addition, BFRT has been postulated to provide benefits not only in muscle mass but also in tendon structure. In fact, some studies have reported similar gains after a resistance training program with low intensities (20–35% 1-RM) combined with BFRT to those reported by high-intensity resistance training (70–85% 1-RM) [8]. A greater resistance to fatigue and a lower perception of effort have also been reported after a period of training with this methodology [9]. Indeed, the ideal occlusive pressure to maximize these improvements is the greatest challenge of BFRT research nowadays [10]. Despite that the underpinning physiological mechanisms that promote these adaptations after low-intensity resistance exercise training combined with BFRT remain unknown [11], it is highly used in clinical contexts [12].

In addition to these training-induced effects, BFR can acutely affect neuromuscular function. Higher decreases in muscle force and electromyography (EMG) amplitude have been shown during BFRT compared to traditional weight training CON. These results indicate that the fatigue caused by BFRT may be due to a combination of peripheral (enhanced twitch reduction) and central fatigue (maximal isometric voluntary contraction (MVIC) and EMG amplitude reduction) [13]. Moreover, is widely known that no differences were found in the recruitment of type II fibers, with type I fibers being preferentially recruited during BFRT [14]. However, it is also known that corticospinal excitability increases after BFRT, showing higher activation levels in comparison with traditional training [2]. These findings suggests that BFRT may lead to a better neural performance [15], which is usually monitored during training through velocity movement control [16] since it allows load quantification [17], 1-RM estimation [18], and neuromuscular fatigue monitorization [19,20].

Recently, Wilk and colleagues [21] showed that BFRT combined with low-intensity resistance exercise (20 to 50% of the estimated 1-RM) led to higher peak velocities during the bench press exercise in comparison with the control condition (i.e., weight training without occlusion). In addition, they observed similar mean concentric velocities between both conditions. This appears to indicate that neuromuscular function is not affected by BFRT when low loads are used, despite contradictory results. However, to the best of our knowledge, it is unknown whether BFRT can lead to less excitation of the involved musculature and to greater intra- and inter-set neuromuscular fatigue. Therefore, this study aimed to analyze the effects of BFRT on neuromuscular performance (i.e., movement velocity and effort index (EI)) and muscle activity in well-trained healthy participants. We hypothesized that the application of BFRT will result in similar kinematic parameters but with some differences in the muscle activity and EI during the back squat exercise.

## 2. Materials and Methods

### 2.1. Participants

Twenty-one healthy volunteers participated in this study, with ten males (aged 25.0 ± 1.5 years) and eleven females (aged 24.4 ± 2.3 years). All participants had no history of lower limb injuries in the past six months and had at least two years of resistance training experience. In addition to the PARQ questionnaire, all participants underwent a physical examination and were assessed through a physical activity habits questionnaire (IPAQ) [22]. The participants were told to avoid taking pain relievers (6 h before), alcohol (48 h before), caffeine (6 h before), and strenuous exercise (48 h before) each session. They were also informed about the general experimental procedures, potential risks, and the research objectives and hypotheses. The research protocol was reviewed and approved by the University Ethics Committee (reference number: EADECAFYD2020-3) in accordance with the ethical guidelines of the Helsinki declaration (World Medical Association Declaration of Helsinki, 2018) [23].

### 2.2. Trial Design

**Figure 1:**
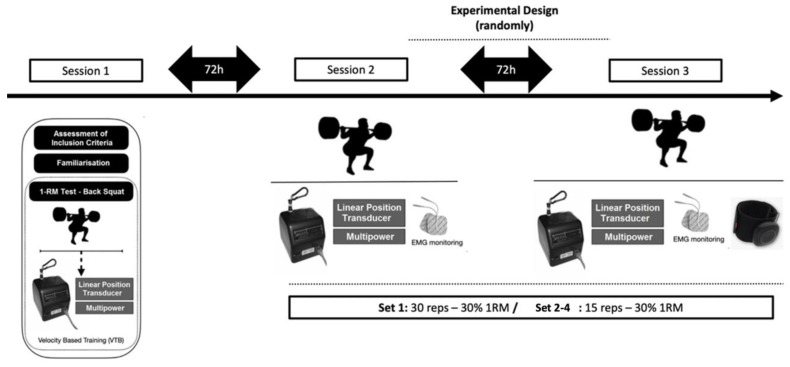
Diagram of study participants and experimental design.

A randomized crossover study was performed to examine the impact of BFRT on muscle-strength-related variables. The participants visited the laboratory three separate times. During their first visit, their height (SECA 220, Hamburg, Germany) and body composition (Tanita RD-545, Tokyo, Japan) were measured, and they underwent a warm-up and a back squat 1-RM test. The second and third visits involved either the BFRT or control (non-BFRT) protocols in a randomized crossover design, with a 72 h break in between (Figure 1).

### 2.3. Procedures

#### 2.3.1. Back Squat One-Repetition Maximum Test

After preparing with a warm-up routine that involved 5 min of cycling at a comfortable pace on a stationary bicycle (Technogym, Gambettola, Forli-Cesena, Italy), 5 min of exercises to mobilize lower limb joints, 3 sets of 30 m sprints, and 3 sets of 5 half squats using weights of 20, 30, and 40 kg, the 1-RM test was conducted on a multipower device (Technogym, Gambettola, Forli-Cesena, Italy) using a progressive loading protocol [17].

The protocol that involves gradual changes in velocity as the load increases was previously described [24]. The best repetition at each weight was selected based on the mean propulsion velocity (MPV) [25]. In addition, the EI as an indicator of accumulated fatigue and perception of effort was measured for both BFRT and non-BFRT conditions according to previous studies [20].

#### 2.3.2. Back Squat Exercise Protocol

The back squat exercise was performed using the same multipower device (Technogym 2017, Gambettola, Forli-Cesena, Italy) that was utilized during the 1-RM test. A highly used protocol to prescribe BFRT was used [26]. It consisted of 4 sets of 30, 15, 15, and 15 repetitions performed by each participant with 60 s rest between sets [26]. The participants started the exercise standing straight, with their knees and hips fully extended (0 degrees knee flexion), feet positioned at shoulder width apart, and both feet flat on the ground parallel to each other. They were instructed to perform each repetition with maximum effort. Encouragement and feedback on velocity were given to motivate participants to put forth their best effort during each repetition. They were required to keep their feet on the ground, but lifting their heels at the end of the lifting phase was allowed because when seeking maximum running speed at relatively low loads (30% RM), limiting this could prevent generating maximum movement velocity. The same warm-up routine used before the 1-RM test was also used before each experimental session.

MPV and peak velocity (Vmax) for the concentric phase of the movement were collected from a linear position transducer (1000 Hz sampling rate (SmartCoach Europe AB, Stockholm, Sweden; SC)). The linear position transducer was the criterion measure to calculate [24]. It was attached to the barbell that participants used to perform the back squat exercise and interfaced with a personal computer for digital data acquisition. During each lift, instantaneous kinematic data were collected with a sample frequency of 1000 Hz. Previous research reported the highly reliable data of the linear position transducer used [27].

#### 2.3.3. BFRT Protocol

During the BFR session, participants wore pressure cuffs at the most proximal region of each leg. The cuffs used were Airbands (Vald Performance, Albion, Australia) with dimensions of 45–64 cm. To establish the specific pressure value, after a 5 min rest, the value for full arterial occlusion pressure was determined automatically by the device. The cuff pressure was controlled at all times by the researcher and monitored through the Vald Performance app^®^. The exercise protocol prescribed 80% of the individual arterial occlusion pressure (AOP), as previously used [28], to enhance the maximum strength and muscle thickness. The cuffs were inflated before starting the exercise.

#### 2.3.4. Surface EMG Protocol

The EMG signals were collected from the dominant leg’s quadriceps muscles (rectus femoris (RF) and vastus lateralis (VL)). The recommended procedure from the SENIAM project was followed for preparing the skin and placing the electrodes [29]. The hair was shaved, and the skin cleaned with alcohol, then rubbed until a reddish color appeared to improve electrode adherence. The same researcher was always in charge of mounting the electrodes to ensure the same location of the electrodes. The electrodes were placed with the subject seated and the knee slightly bent (distance between electrodes: 20 mm), and data were collected using a wireless EMG system (mDurance Solutions SL, Granada, Spain) with 4 EMG channels, a sampling frequency of 1024 Hz, and a bandwidth of 8.4 kHz. The resolution of the EMG signal was 24 bits, and the amplification was 100–10,000 V/V. The electrodes were pre-gelled Ag/AgCl with a diameter of 10 mm, the location of each motor point was determined using a low-voltage motor point pencil unit [30], and the location was marked to ensure that both measurements were taken at the same point [31]. A reference electrode was placed on the head of the same leg’s fibula [32].

After electrode placement, participants sat down and performed a maximum voluntary isometric contraction (MVIC) of the quadriceps with the knee at 90° [30]. Two repetitions of the isometric knee extension test were performed for 5 s, separated by a rest period of 2 min [33]. Participants were instructed to gradually increase the force of the muscle contraction for up to a maximum of 2 s, holding the resulting MVIC for 3 s [34]. The root mean square EMG (EMGrms) signal was recorded.

Tests were filtered using a fourth order Butterworth bandpass filter with a cut-off frequency at 20–450 Hz. The signal was smoothed using a window size of 0.025 s RMS and an overlapping of 0.0125 s between windows [35]. Before the start of the back squat test in each of the sessions, the basal level of the EMG signal was monitored to check that there were no differences in this value.

#### 2.3.5. Statistical Analysis

All statistical analysis was performed using the Jamovi software package (The Jamovi Project, v.1.6.23.0) Normality was checked using the Shapiro–Wilk normality test. Then, a repeated measures linear mixed model fitted with a restricted maximum likelihood method and unstructured covariates was used to compare outcomes between sets (sets 1–4) and conditions (BFRT and non-BFRT). The main outcomes used in statistical analyses were peak and mean concentric velocity and EMG_RMS_ and peak normalized EMG activation for the RF and VL muscles. The level of significance for all tests was set to *α* = 0.05. Mean, standard deviation (SD), and the t value were reported for all statistical analyses.

Sample size was estimated for a repeated measures ANOVA using G*power (G*Power 3.1.9.2, Heinrich Heine-Universitat Dusseldorf, Dusseldorf, Germany). The effect size was computed using the means and between-subject SDs from a previously published study [36] that analyzed kinematic data during the back squat exercise with and without BFR. The means and SDs were 1.57 ± 0.12 and 1.45 ± 0.15 m·s^−1^ of peak concentric velocity, respectively. The 95% confidence intervals were therefore 1.58–1.66 and 1.35–1.56 m·s^−1^, respectively, resulting in a Cohens *d_z_* effect size of 0.88, which can be classified as, and so equivalent to, an f = 0.3 (moderate). The average SD was used to compute the effect size. Alpha was set to 5%, while power was set at 80% (1 − *β*). The estimated sample size was 12 participants by sex (actual power = 0.846), but considering possible dropouts, and therefore 24 participants were enrolled in this study.

## 3. Results

Twenty-four healthy volunteers participated in this study. Three participants (one male and two females) dropped out of the study due to personal reasons, leaving twenty-one participants (male (*n* = 10): 25.0 ± 1.5 years, 178 ± 7.6 cm, 76.8 ± 11.2 kg, 26.8 ± 4.2 kg·m^−2^; female (*n* = 11): 24.4 ± 2.3 years, 168 ± 3.6 cm, 61.3 ± 6.4 kg, 21.6 ± 3.9 kg·m^−2^) who completed the study protocol.

Regarding movement velocity, significant effects were only observed for the exercise set (*p* < 0.001, F = 13.1; i.e., Set 1 vs. Set 2: *p* < 0.001, t = 4.2; and Set 1 vs. Set 3: *p* < 0.001, t = 4.1) for EI. However, no significant interactions were observed between exercise set and condition (*p* = 0.718, F = 0.45). No significant interactions between sets, conditions, and set*condition were observed for MPV and Vmax. As shown in Table 1, Set 1 in the BFRT condition manifested a higher (*p* = 0.033) EI compared with Set 2 (mean difference (SD and t): 8.57 (SD = 2.5, t = 3.5)). Similarly, Set 1 in the BFRT condition showed a higher (*p* = 0.037) EI compared with Set 3 in the non-BFRT condition (mean difference: 9.96 (SD = 2.9, t = 3.5)). Set 2, Set 3, and Set 4 did not show significant differences between them in the BFRT condition nor in the non-BFRT condition. No between-group differences were observed for any set. Regarding peak and mean concentric velocity, similar results were observed (Table 1).

An individual analysis of the EMGmax and EMGrms of each muscle was performed (Table 1). Regarding RF, significant effects for exercise set in EMGmax (*p* = 0.003, F = 5.0; Set 1 vs. Set 2: *p* < 0.001, t = 4.6) and EMGrms (*p* < 0.001, F = 12.6; Set 1 vs. Set 2: *p* < 0.001, t = 4.9; and Set 1 vs. Set 3; *p* = 0.002, t = 4.0) were observed. As shown in Table 1, higher RF EMGmax (9.3 μV, *p* = 0.004, SD = 2.2, t = 4.2) and EMGrms (9.3 μV, *p* = 0.05, SD = 23.3, t = 2.7) values were observed when comparing Set 1 and Set 2 in the BFRT condition. However, no other significant interactions between sets or between conditions were observed. Similarly, regarding VL, significant effects were shown for exercise set in EMGmax (*p* = 0.002, F = 5.4; Set 1 vs. Set 2: *p* = 0.005, t = 3.6; and Set 1 vs. Set 3; *p* = 0.042, t = 2.8) and EMGrms (*p* < 0.001, F = 7.0; Set 1 vs. Set 2: *p* = 0.01, t = 3.4; and Set 1 vs. Set 3; *p* = 0.042, t = 2.8) were observed. However, the results were similar between series and between conditions. No significant differences were observed. 

## 4. Discussion

This study aimed to analyze the effects of BFRT with 80% of AOP on kinematic and EMG parameters during low-intensity (i.e., 30% of 1-RM) back squat exercise. The main result of this study was that no significant differences in MPV and Vmax were observed between sets of the back squat exercise during both BFRT and non-BFRT conditions. However, higher intra-set EIs were observed in Set 1 compared to Set 2 when BFRT was applied. Similarly, higher RF, EMGpeak, and EMGrms were observed in Set 1 compared to Set 2 after BFRT. Therefore, BFRT did not result in significant changes in neuromuscular performance during low-intensity resistance training. However, it may lead to greater intra-set velocity loss and lower activation of involved musculature when high volumes (i.e., more than 15 repetitions) were prescribed but not when lower volumes (i.e., up to 15 repetitions) were used.

To date, only a few studies have investigated the acute effects of BFRT on movement velocity during the back squat exercise [37,38]. Recent studies [39] compared different AOPs (from 40 to 100%) during the squat and bench press exercises performed at 60% 1-RM. They showed an increase in MPV during the squat with 100% of AOP. Similarly, another study [40] showed that the back squat exercise performed at 70% 1-RM with 150% of AOP had a significantly higher peak concentric velocity and power output. On the other hand, as was observed in our results, when lower cuff pressure (less AOP than 100%) was applied, no differences were observed between BFRT and traditional resistance exercise on kinematic parameters regardless of load used (30% 1-RM). According to previous studies [41,42], it seems that the higher the AOP, the greater the enhancements in kinematic parameters during multi-joint exercises. The EI together with the EMG allow us to know the real degree of effort made during a strength exercise. The results of our study provide valuable information for comparing and equating the effort made in the different interventions. The main contribution of a velocity-based RT approach is that it provides the necessary information to know the actual training loads that induce a specific effect in each athlete, and the EI gives us better knowledge about the acute and chronic effects of a given training design like BFRT, showing the muscle fatigue induced by an specific exercise [16].

Therefore, using a lower AOP in combination with low loads during BFRT could be an effective strategy to enhance performance without increasing intensity or AOP, as our results demonstrated. Therefore, implementing low-intensity BFRT could be an interesting approach for rehabilitation purposes or any other scenario where it is not possible to prescribe high-intensity training.

BFRT has been usually prescribed with low-intensity exercises [40]. Indeed, some studies [41,42] have shown that BFRT with heavy loads did not lead to additional benefits. In addition, low loads (between 20 and 50% 1-RM) are used because they do not compromise performance during exercise. Yasuda et al. [43] showed that low-intensity strength training and BFR-induced functional muscle adaptations are enhanced when combined with HI-RT. Even so, the use of low-intensity BFRT has shown benefits in increasing lower limb strength in rehabilitation processes, especially in middle-aged people [44].

Another aspect to take into account when using this technique is the sequence of application. A study [21] that combined continuous and intermittent BFRT using both low and high intensities showed that BFRT used during resistance exercise training increases peak concentric velocity, which in turn validates this method for performance purposes (i.e., enhanced explosiveness). In addition, it has been proposed that BFRT using loads above 60% 1-RM leads to higher intramuscular pressure due to increased mechanical tension, which results in stemmed blood flow during exercise [45]. Additionally, blood flow during recovery can be compromised due to muscle inflammation as a result of osmotic and fluid changes that occur in the muscle [46,47,48]. Therefore, since blood flow during exercise and recovery is reduced due to muscle tension and inflammation, as it occurs with heavy weightlifting, additional hypoxia induced by BFRT does not cause further metabolic effects [21]. Thus, low loading patterns during BFRT are usually employed to enhance the metabolic response that we could achieve at the same intensity with traditional exercises [3]. This increased metabolic response and the acidic environment promoted by hypoxia greatly influences muscle protein synthesis, altering the genetic regulation of satellite muscle cells, increasing muscle fiber recruitment, and improving muscle endurance [3]. Hypoxia and the accumulation of metabolites are thought to promote muscle fiber recruitment. The activation of fast-twitch fibers is essential for improving athletic performance. Several studies that measure muscle activation through EMG during non-fatigued contractions have shown significantly greater responses with the application of BFRT [49]. However, other studies [42] did not show an increase in EMG activity during low-load BFRT compared to traditional heavy-load RT. These results are in line with our data, as no differences were observed between the BFRT condition and non-BFRT condition in muscle activity. In our case, we have observed that performing a high-volume exercise (i.e., number of repetitions) can affect the excitation of the involved muscles in the subsequent set (Table 1). Therefore, another aspect that should be further considered is the effect of volume and neuromuscular fatigue on muscle activation since it can influence the effects of BFRT. Acute fatigue is usually associated with specific factors of a peripheral source related to the acidity of the intramuscular environment, causing failures in the neuromuscular junction mechanism [50]. Thus, a decrease in the amplitude of the electromyography signals during the series of exercise appears to indicate the beginning of fatigue when associated with a shortage in the capacity of muscle strength. These aspects should be taken into account when prescribing exercise load to athletes and patients applying BFRT.

Another important aspect to consider in the application of BFRT is the possible differences in applying it only in a single training session or over a longer period. There are limited studies that have specifically compared the acute and chronic effects of BFRT. A study [51] showed that two sessions per day of training with the same volume do not necessarily result in larger responses in all hormones than one session per day of training. Another study found, after applying a 7-week intervention with BFRT and loads at 20% of 1-RM, similar magnitudes and mechanisms for strength adaptation and intramuscular anabolic activity as those found with a heavy load (70% 1-RM). Similarly, and with applicability in performance sports, the periodic restriction of local blood flow followed by reperfusion has been shown to improve performance in cycling, running, and swimming [52]. In relation to chronic effects, ischemic preconditioning has been proposed as a possible explanation for the increase in improvement in sports performance with a high AOP [53]. After a brief ischemia produced during a certain period of time, they can perform an ergogenic effect because the hyperemia experienced after the occlusion seems to produce an increase in the production of nitric oxide, in addition to an increase in the resynthesis of phosphocreatine, an altered kinetics of oxi-desoxyhemoglobin, and a greater oxygen consumption [54,55]. Therefore, it seems that applying BFRT with low intensity could be effective and similar to training with high loads without restriction and that both acute and chronic effects could be obtained using this methodology. Specially, the results of this study are interesting in both a sports performance and rehabilitation context when lifting heavy training loads is contraindicated.

## 5. Limitations

Our study presented some concerns that should be noted. This study might be considered as an exploratory study due to the low sample size (21 participants); however, the calculation of statistical power helped to draw accurate conclusions in the population used. Therefore, readers should interpret and generalize our results with caution since further research is needed to provide an accurate and reliable BFRT application pathway. Moreover, we cannot extrapolate our results to patients undergoing rehabilitation or physical reconditioning, as only healthy and well-trained participants volunteered in this study. Finally, more studies are warranted to understand the time-course effects and chronic adaptations on neuromuscular performance of velocity-based BFRT.

## 6. Conclusions

In conclusion, low-intensity BFRT performed with 80% AOP did not induce changes in neuromuscular performance (kinematic and EMG parameters) during the back squat exercise in well-trained young men and women. However, high volumes (i.e., more than 15 repetitions) induced greater intra-set velocity loss and lower excitation of the involved musculature compared to lower volumes. Therefore, the implementation of BFRT is an effective and safe strategy in healthy populations to improve their performance without increasing training intensity.

## Figures and Tables

**Table 1:** Mean ± standard deviation (minimum–maximum) of velocity and EMG outcomes for each set.

	BFRT Condition (*n* = 21)				Non-BFRT Condition (*n* = 21)			
	Set 1	Set 2	Set 3	Set 4	Set 1	Set 2	Set 3	Set 4
**Movement velocity**								
MPV (m·s^−1^)	0.85 ± 0.11 (0.70–1.05)	0.85 ± 0.10 (0.69–1.06)	0.85 ± 0.11 (0.67–1.07)	0.85 ± 0.11 (0.63–1.05)	0.85 ± 0.11 (0.65–1.02)	0.85 ± 0.11 (0.62–1.07)	0.84 ± 0.12 (0.61–1.05)	0.86 ± 0.12 (0.63–1.11)
Vmax (m·s^−1^)	1.39 ± 0.17 (1.11–1.71)	1.37 ± 0.16 (1.10–1.67)	1.36 ± 0.17 (1.10–1.69)	1.38 ± 0.18 (1.04–1.71)	1.41 ± 0.17 (1.13–1.66)	1.39 ± 0.18 (1.03–1.75)	1.39 ± 0.20 (1.01–1.73)	1.38 ± 0.20 (1.08–1.85)
EI	20.7 ± 14.6 *,^ (6.6–58.7)	12.1 ± 7.4 (3.9–32.1)	13.1 ± 6.0 (3.4–26.1)	12.5 ± 6.0 (3.9–23.9)	17.3 ± 9.2 (6.6–48.4)	11.2 ± 3.9 (4.2–20.5)	10.7 ± 3.9 (6.2–20.6)	11.9 ± 6.3 (6.0–30.1)
**Muscle activity**								
RF EMGmax (μV)	82.1 ± 19.8 * (29.0–102.0)	72.8 ± 16.6 (29.4–100.0)	73.8 ± 17.2 (26.3–97.8)	74.8 ± 18.3 (26.5–104.0)	77.0 ± 18.8 (25.4–102.0)	72.0 ± 17.0 (24.7–99.4)	75.9 ± 19.7 (24.8–100.9)	73.7 ± 18.0 (24.0–100.0)
RF EMGrms (%)	34.9 ± 9.1 * (15.1–46.1)	29.2 ± 6.9 (16.6–46.4)	29.7 ± 6.8 (13.8–40.0)	30.1 ± 6.4 (17.2–40.6)	34.4 ± 11.9 (0.3–49.2)	30.4 ± 8.0 (10.5–48.3)	30.0 ± 8.6 (11.4–48.6)	30.4 ± 8.3 (10.1–48.3)
VL EMGmax(μV)	83.3 ± 18.0 (44.9–110.4)	79.9 ± 17.0 (43.9–100.0)	79.7 ± 16.6 (43.9–99.3)	78.8 ± 16.3 (41.7–100.2)	84.7 ± 15.0 (46.8–103.0)	78.9 ± 13.4 (44.2–95.9)	80.5 ± 11.3 (50.3–101.1)	78.6 ± 12.2 (52.5–100.1)
VL EMGrms (%)	39.7 ± 8.3 (22.8–56.2)	35.5 ± 7.6 (23.2–51.0)	36.1 ± 7.4 (22.9–48.3)	36.5 ± 8.2 (21.0–52.6)	39.9 ± 11.9 (0.5–53.0)	36.3 ± 6.9 (18.7–50.7)	35.8 ± 6.8 (18.6–50.1)	36.1 ± 7.2 (20.7–50.7)

Note: Values are means ± standard deviation. Abbreviations: EI: effort index; EMGmax: peak electromyographic activation; MPV: mean propulsive velocity; RF: rectus femoris; EMGrms: root mean square normalized electromyographic activation; VL: vastus lateralis; Vmax: peak concentric velocity. * Significant (*p* < 0.05) difference from Set 2 in BFRT. ^ Significant (*p* < 0.05) difference from Set 3 in non-BFRT.

## Data Availability

Not applicable.

## References

[1] Ferraz R.B., Gualano B., Rodrigues R., Kurimori C.O., Fuller R., Lima F.R., De Sá-Pinto A.L., Roschel H. (2018). Benefits of Resistance Training with Blood Flow Restriction in Knee Osteoarthritis. Med. Sci. Sports Exerc..

[2] Kjeldsen S.S., Næss-Schmidt E.T., Hansen G.M., Nielsen J.F., Stubbs P.W. (2019). Neuromuscular effects of dorsiflexor training with and without blood flow restriction. Heliyon.

[3] Bowman E.N., Elshaar R., Milligan H., Jue G., Mohr K., Brown P., Watanabe D.M., Limpisvasti O. (2019). Proximal, Distal, and Contralateral Effects of Blood Flow Restriction Training on the Lower Extremities: A Randomized Controlled Trial. Sports Health.

[4] Labata-Lezaun N., Llurda-Almuzara L., González-Rueda V., López-de-Celis C., Cedeño-Bermúdez S., Bañuelos-Pago J., Perez-Bellmunt A. (2022). Effectiveness of Blood Flow Restriction Training on Muscle Strength and Physical Performance in Older Adults: A Systematic Review and Meta-analysis. Arch. Phys. Med. Rehabil..

[5] Maga M., Wachsmann-Maga A., Batko K., Włodarczyk A., Kłapacz P., Krężel J., Szopa N., Sliwka A. (2023). Impact of Blood-Flow-Restricted Training on Arterial Functions and Angiogenesis—A Systematic Review with Meta-Analysis. Biomedicines.

[6] Pant G., Bhutia U.D. (2017). Effect of restricted blood flow on muscle hypotrophy & O2 saturation level on weight training. Int. J. Phys. Educ. Sport. Health.

[7] Flocco P., Galeoto G. (2021). Effect of blood flow restriction training on physiological outcomes in healthy athletes: A systematic review and meta-analysis. Muscles Ligaments Tendons J..

[8] Centner C., Jerger S., Lauber B., Seynnes O., Friedrich T., Lolli D., Gollhofer A., König D. (2021). Low-Load Blood Flow Restriction and High-Load Resistance Training Induce Comparable Changes in Patellar Tendon Properties. Med. Sci. Sports Exerc..

[9] Schwiete C., Franz A., Roth C., Behringer M. (2021). Effects of Resting vs. Continuous Blood-Flow Restriction-Training on Strength, Fatigue Resistance, Muscle Thickness, and Perceived Discomfort. Front Physiol..

[10] Wortman R.J., Brown S.M., Savage-Elliott I., Finley Z.J., Mulcahey M.K. (2021). Blood Flow Restriction Training for Athletes: A Systematic Review. Am. J. Sports Med..

[11] Yasuda T., Loenneke J.P., Thiebaud R.S., Abe T. (2012). Effects of Blood Flow Restricted Low-Intensity Concentric or Eccentric Training on Muscle Size and Strength. PLoS ONE.

[12] Chulvi-Medrano I., Picón-Martínez M., Cortell-Tormo J.M., Tortosa-Martínez J., Alonso-Aubin D.A., Alakhdar Y. (2021). Different time course of recovery in achilles tendon thickness after low-load resistance training with and without blood flow restriction. J. Sport Rehabil..

[13] Karabulut M., Cramer J.T., Abe T., Sato Y., Bemben M.G. (2010). Neuromuscular fatigue following low-intensity dynamic exercise with externally applied vascular restriction. J. Electromyogr. Kinesiol..

[14] Centner C., Ritzmann R., Schur S., Gollhofer A., König D. (2019). Blood flow restriction increases myoelectric activity and metabolic accumulation during whole-body vibration. Eur. J. Appl. Physiol..

[15] Queiros V.S., De França I.M., De Trybulski R. (2021). Myoelectric Activity and Fatigue in Low-Load Resistance Exercise with Different Pressure of Blood Flow Restriction: A Systematic Review and Meta-Analysis. Front Physiol..

[16] González-Badillo J.J., Sánchez-Medina L., Ribas-Serna J., Rodríguez-Rosell D. (2022). Toward a New Paradigm in Resistance Training by Means of Velocity Monitoring: A Critical and Challenging Narrative. Sport Med—Open.

[17] García-Ramos A., Suzovic D., Pérez-Castilla A. (2019). The load-velocity profiles of three upper-body pushing exercises in men and women. Sport Biomech..

[18] Sánchez-Medina L., Pallarés J., Pérez C., Morán-Navarro R., González-Badillo J. (2017). Estimation of Relative Load From Bar Velocity in the Full Back Squat Exercise. Sport Med. Int. Open.

[19] Sánchez-Medina L., González-Badillo J.J. (2011). Velocity loss as an indicator of neuromuscular fatigue during resistance training. Med. Sci. Sports Exerc..

[20] Rodríguez-Rosell D., Yáñez-García J.M., Torres-Torrelo J., Mora-Custodio R., Marques M.C., González-Badillo J.J. (2018). Effort index as a novel variable for monitoring the level of effort during resistance exercises. J. Strength Cond. Res..

[21] Wilk M., Gepfert M., Krzysztofik M., Stastny P., Zajac A., Bogdanis G.C. (2020). Acute Effects of Continuous and Intermittent Blood Flow Restriction on Movement Velocity During Bench Press Exercise Against Different Loads. Front Physiol..

[22] Cancela J., Ayán C., Vila H., Gutiérrez J., Gutiérrez-Santiago A. (2019). Validez de Constructo del Cuestionario Internacional de Actividad Física en Universitarios Españoles. Rev. Iberoam. Diagnóstico Evaluación Avaliação Psicológica.

[23] Shrestha B., Dunn L. (2020). The Declaration of Helsinki on Medical Research involving Human Subjects: A Review of Seventh Revision. J. Nepal. Health Res. Counc..

[24] Sanchez-Medina L., Perez C.E., Gonzalez-Badillo J.J. (2010). Importance of the propulsive phase in strength assessment. Int. J. Sports Med..

[25] Schoenfeld B.J., Pope Z.K., Benik F.M., Hester G.M., Sellers J., Nooner J.L., Schnaiter J.A., Bond-Williams K.E., Carter A.S., Ross C.L. (2016). Longer interset rest periods enhance muscle strength and hypertrophy in resistance-trained men. J. Strength Cond. Res..

[26] Garcia-Sillero M., Chulvi-Medrano I., Maroto-Izquierdo S., Bonilla D.A., Vargas-Molina S., Benítez-Porres J. (2022). Effects of Preceding Transcranial Direct Current Stimulation on Movement Velocity and EMG Signal during the Back Squat Exercise. J. Clin. Med..

[27] García-Sillero M., Jurado-Castro J.M., Benítez-Porres J., Vargas-Molina S. (2021). Acute effects of a percussive massage treatment on movement velocity during resistance training. Int. J. Environ. Res. Public Health.

[28] Pope Z.K., Willardson J.M., Schoenfeld B.J. (2013). Exercise and blood flow restriction. J. Strength Cond. Res..

[29] Stegeman D., Hermens H. (2007). Standards for Surface Electromyography: The European Project Surface EMG for Non-Invasive Assessment of Muscles (SENIAM). Enschede: Roessingh Res. Dev..

[30] Hermens H.J., Merletti R., Rix H., Freriks B. (1998). The State of the Art on Signal Processing Methods for Surface ElectroMyoGraphy. Seniam.

[31] Gobbo M., Maffiuletti N.A., Orizio C., Minetto M.A. (2014). Muscle motor point identification is essential for optimizing neuromuscular electrical stimulation use. J. Neuroeng. Rehabil..

[32] Napoli N.J., Mixco A.R., Bohorquez J.E., Signorile J.F. (2015). An EMG comparative analysis of quadriceps during isoinertial strength training using nonlinear scaled wavelets. Hum. Mov. Sci..

[33] Park J., Ty Hopkins J. (2011). Quadriceps activation normative values and the affect of subcutaneous tissue thickness. J. Electromyogr. Kinesiol..

[34] Roberts D., Kuenze C., Saliba S., Hart J.M. (2012). Accessory muscle activation during the superimposed burst technique. J. Electromyogr. Kinesiol..

[35] Molina-Molina A., Ruiz-Malagón E.J., Carrillo-Pérez F., Roche-Seruendo L.E., Damas M., Banos O., García-Pinillos F. (2020). Validation of mDurance, A Wearable Surface Electromyography System for Muscle Activity Assessment. Front. Physiol..

[36] Jakobsen M.D., Sundstrup E., Andersen C.H., Aagaard P., Andersen L.L. (2013). Muscle activity during leg strengthening exercise using free weights and elastic resistance: Effects of ballistic vs controlled contractions. Hum. Mov. Sci..

[37] Gepfert M., Krzysztofik M., Kostrzewa M., Jarosz J., Trybulski R., Zajac A., Wilk M. (2020). The acute impact of external compression on back squat performance in competitive athletes. Int. J. Environ. Res. Public Health.

[38] Wilk M., Trybulski R., Krzysztofik M., Wojdala G., Campos Y., Zajac A., Lulińska E., Stastny P. (2021). Acute Effects of Different Blood Flow Restriction Protocols on Bar Velocity During the Squat Exercise. Front. Physiol..

[39] Serrano-Ramon J., Cortell-Tormo J., Bautista I., García Jaén M., Chulvi-Medrano I. (2023). Acute effects of different external compression with blood flow restriction on force-velocity profile during squat and bench press exercises. Biol. Sport.

[40] Farup J., de Paoli F., Bjerg K., Riis S., Ringgard S., Vissing K. (2015). Blood flow restricted and traditional resistance training performed to fatigue produce equal muscle hypertrophy. Scand. J. Med. Sci. Sport.

[41] Teixeira E.L., Painelli V de S., Schoenfeld B.J., Silva-Batista C., Longo A.R., Aihara A.Y., Cardoso F.N., Peres B.D.A., Tricoli V. (2020). Perceptual and Neuromuscular Responses Adapt Similarly Between High-Load Resistance Training and Low-Load Resistance Training with Blood Flow Restriction. J. Strength Cond. Res..

[42] Laurentino G., Ugrinowitsch C., Aihara A.Y., Fernandes A.R., Parcell A.C., Ricard M., Tricoli V. (2008). Effects of strength training and vascular occlusion. Int. J. Sports Med..

[43] Yasuda T., Ogasawara R., Sakamaki M., Ozaki H., Sato Y., Abe T. (2011). Combined effects of low-intensity blood flow restriction training and high-intensity resistance training on muscle strength and size. Eur. J. Appl. Physiol..

[44] Chang H., Yao M., Chen B., Qi Y., Zhang J. (2022). Effects of Blood Flow Restriction Combined with Low-Intensity Resistance Training on Lower-Limb Muscle Strength and Mass in Post-Middle-Aged Adults: A Systematic Review and Meta-Analysis. Int. J. Environ. Res. Public Health.

[45] Gołaś A., Maszczyk A., Petr M., Statsny P., Wilk M., Wróbel G. (2015). Changes in Bar Velocity and Muscular Activity during the Bench Press in Relation to the Load Lifted. Cent. Eur. J. Sport Sci. Med..

[46] Gabriel D.A., Kamen G., Frost G. (2006). Neural adaptations to resistive exercise: Mechanisms and recommendations for training practices. Sport Med..

[47] Moore D.R., Burgomaster K.A., Schofield L.M., Gibala M.J., Sale D.G., Phillips S.M. (2004). Neuromuscular adaptations in human muscle following low intensity resistance training with vascular occlusion. Eur. J. Appl. Physiol..

[48] Neto G.R., Santos H.H., Sousa J.B.C., Júnior A.T.A., Araújo J.P., Aniceto R.R., Sousa M.S.C. (2014). Effects of high-intensity blood flow restriction exercise on muscle fatigue. J. Hum. Kinet..

[49] Takarada Y., Takazawa H., Sato Y., Takebayashi S., Tanaka Y., Ishii N. (2000). Effects of resistance exercise combined with moderate vascular occlusion on muscular function in humans. J. Appl. Physiol..

[50] Gandevia S.C. (2001). Spinal and supraspinal factors in human muscle fatigue. Physiol. Rev..

[51] Sharifi S., Monazzami A., Nikousefat Z., Heyrani A., Yari K. (2020). The acute and chronic effects of resistance training with blood flow restriction on hormonal responses in untrained young men: A comparison of frequency. Cell Mol. Biol..

[52] Incognito A.V., Burr J.F., Millar P.J. (2016). The Effects of Ischemic Preconditioning on Human Exercise Performance. Sport Med..

[53] de Souza H.L.R., Arriel R.A., Hohl R., da Mota G.R., Marocolo M. (2021). Is Ischemic Preconditioning Intervention Occlusion-Dependent to Enhance Resistance Exercise Performance?. J. Strength Cond. Res..

[54] Bailey T.G., Birk G.K., Timothy Cable N., Atkinson G., Green D.J., Jones H., Thijssen D.H.J. (2012). Remote ischemic preconditioning prevents reduction in brachial artery flow-mediated dilation after strenuous exercise. Am. J. Physiol. Hear. Circ. Physiol..

[55] Andreas M., Schmid A.I., Keilani M., Doberer D., Bartko J., Crevenna R., Moser E., Wolzt M. (2011). Effect of ischemic preconditioning in skeletal muscle measured by functional magnetic resonance imaging and spectroscopy: A randomized crossover trial. J. Cardiovasc. Magn. Reson..

